# Ethnicity Influences Corpus Callosum Dimensions

**DOI:** 10.1155/2018/8916035

**Published:** 2018-05-02

**Authors:** Hilda Nouri Hosseini, Mohammad Reza Mohammadi, Mohsen Aarabi, Narges Mohammadi, Mohammad Jafar Golalipour

**Affiliations:** ^1^5 Azar Hospital, Golestan University of Medical Sciences, Gorgan, Iran; ^2^Department of Neurosurgery, Golestan University of Medical Sciences, Gorgan, Iran; ^3^Department of Epidemiology, Mazandaran University of Medical Sciences, Sari, Iran; ^4^Gorgan Congenital Malformations Research Center, Golestan University of Medical Sciences, Gorgan, Iran; ^5^Gorgan Congenital Malformations Research Center, Department of Anatomical Sciences, Golestan University of Medical Sciences, Gorgan, Iran

## Abstract

**Background and Objective:**

Corpus callosum (CC), the main white matter cable which connects two hemispheres of brain, is important in special procedures such as stereotaxic surgeries vary in size, in different populations. Determination of possible size differences in ethnical groups has special values.

**Patients and Methods:**

The size of the CC on midsagittal view was determined in 76 normal male subjects using MRI of brain hemispheres in northern Iran. The size of rostrum, body, splenium, length, and height of CC was measured for each subject. The width of the body of the corpus callosum (*B*), the anterior to posterior length (*L*) and the maximum height (*H*) of the corpus callosum, and ratios *B*/*L* and *B*/*H* were also calculated.

**Results:**

The longitudinal dimensions of the CC were 70.21 mm and 74.05 mm in native Fars and Turkmens, respectively (*P* < 0.05). The heights were 25 mm and 25.75 mm in native Fars and Turkmen subjects, respectively. The width of CC in Turkmen people was significantly higher than native Fars people (*P* < 0.05). The Evans index in Turkmen group (0.314) was significantly higher than in native Fars (0.3). The *B*/*L* and *B*/*H* ratios were nonsignificantly different between two groups.

**Conclusion:**

The CC parameters vary in different ethnical groups in northern Iran.

## 1. Background

Corpus callosum the major interhemispheric commisure connects two brain hemispheres [1]. Corpus callosum has the main role in language, prosody, and functional connection between the motor and sensory cortices of brain hemispheres [2, 3].

Several diseases, including bipolar disorder [4], Alzheimer [5], Leukoaraiosis [6], and Williams's syndrome [7], can alter the corpus callosum size in human.

Also, morphological alterations of the corpus callosum were reported in some diseases including dyslexia [8], Tourette's syndrome [9], Down's syndrome [10], Depression [11], Schizophrenia [12], and HIV/AIDS [13].

Corpus callosum dimensions seems to be various in different ethnical or racial populations; therefore, determining corpus callosum dimensions and sex-related differences is important in the diagnosis of diseases [14].

Several studies have been performed on the size and shape of the CC of Caucasian population [1, 15–19] and some studies reported in Japanese [20, 21] and Indian populations [22–24] and not in Iranian ones according to race/ethnicity.

Two major ethnic groups (native Fars and Turkmen) are residing in Gorgan, Golestan province in northern Iran; Golestan province has a population of about 1.8 million. Fars group is the predominant inhabitant of this province. The Turkmen people originally are from central Asia who moved here 200 years ago, and because of their special cultural belief, they do not mix with other residential groups. Although several studies have reported the effect of ethnicity on brain size and cranial capacity [25–27], there is no report regarding corpus callosum dimensions according to ethnicity in Iran.

Therefore, this study was carried out to evaluate the dimensions of the corpus callosum depending on the ethnical groups in healthy Iranian population.

## 2. Materials and Methods

This descriptive study was done on 76 (40 native Fars, 36 Turkmen) subjects admitted to the Kowsar MRI Center in Gorgan, northern Iran, from July 2012 to December 2012. Subjects' consent was obtained for the study along with a clearance from the institutional ethical committee.

The subjects consisted of 76 men (range: 35–43 years old) without any brain disorder on MRI, and neurological symptoms and history of drug and drinking were enrolled in the study.

Brain and corpus callosum dimensions were measured on MRI Unit (Siemens, Symphony, 1.5 Tesla). MR images were acquired in the axial and vertical and sagittal planes by using flair, T1, and T2 weighted sequences.

Using a midsagittal section of the cerebral hemispheres, the width of all parts, length, and the height of CC were measured for each subject. For determining the parts of CC the two lines including a line from the inferior borders of the splenium to rostrum and a vertical line extending to the first line were drawn. The width of the body of the corpus callosum (*B*), the anterior to posterior length (*L*) and the maximum height (*H*) of the corpus callosum, and ratios *B*/*L* and *B*/*H* were evaluated.

Also, using axial T1-weighted (TR/TE300/25 ms) images, the Evans index (maximum distance between the two anterior horns/maximum transverse inner diameter of the skull at the same level) and the maximum width of the third ventricle were measured.

The two different persons independently performed measurement and calculation of indices and ratios. All cases were known as numbers and investigators did not have any information about them.

The differences among ethnical groups were evaluated using one-way analysis of variance followed by Fisher's protected least-square difference test. The *P* value less than 5% was considered significant.

## 3. Results

The corpus callosum dimensions according to ethnicity are depicted in Table 1.

In addition, a significant difference of A-P length between males in two groups were seen (*P* < 0.001). Other differences between males in Fars and Turkmen were not statistically significant.

The mean values of the longitudinal dimension of the corpus callosum were 70.21 (95% CI: 68.85–71.58) and 74.05 mm (95% CI: 72.43–75.68) mm in native Fars and Turkmen subjects, respectively (*P* < 0.0001).

The mean values for the height of the corpus callosum were 25 (95% CI: 24.28–25.74) and 25.75 mm (95% CI: 24.79–26.71) mm in native Fars and Turkmen subjects, respectively.

The mean value for the width of the splenium in Turkmen subjects was significantly higher than native Fars subjects (*P* < 0.033).

The mean value for the width of the body of the corpus callosum and the width of the rostrum in Turkmen subjects was nonsignificantly higher than native Fars subjects.

The mean value for Evans index in Turkmen subjects (0.314) was significantly higher than native Fars subjects (0.3) (*P* < 0.001).

The *B*/*L* ratio in native Fars subjects was nonsignificantly higher than Turkmen subjects, but the *B*/*H* ratio in Turkmen subjects was nonsignificantly higher than native Fars subjects.

## 4. Discussion

In recent years, most of the available studies have been carried out on MRI scans in various parts of the world concerning the diameters and morphology differences of corpus callosum [1, 14–24, 28–30].

This study showed evidence for ethnical dimorphism in length of CC, the width of the body, and the width of splenium and Evans index.

In Takeda study, using the MRI method, the length and height of CC were reported 69.7 ± 4.15 and 25.9 ± 2.90 mm in Japanese males, respectively [21].

According to Bermudez and Zatorre study, the total area of CC was significantly larger in men, as we have anterior third and posterior midbody. However, in females, relatively anterior midbody and splenium were larger. According to Bermudez and Zatorre opinion, there was a clear document for regional differences in size and possible shape and position of the CC between the males and females [15].

In Indian males, the length and height of CC were reported as 7.57 cm and 3.27 cm, respectively. Also, the splenium width size was 1.15 cm. Furthermore, length, height, and most of the widths of CC of Indian people were more than the Japanese but the length and width of CC were less than those of Caucasians [23].

Mourgela et al. (2007) in Greece reported that there was a positive linear association between longitudinal and vertical length of the brain and the space of the CC from the frontal and occipital poles of brain hemisphere, although there was no significant correlation between the brain length with the CC length [1].

Lee et al. (2008) reported that the orders of the length of anterior-posterior commissure distance were varied in Caucasian, Asian, Black, and Hispanic populations. According to Lee's findings, the racial factor can significantly affect the AC-PC distance [31].

Several studies reported that only longitudinal dimension of CC is higher in males [1, 24].

In this study, longitudinal dimensions of CC were more than other studies [1, 21, 24]. Also, the width of CC was similar to other studies [1, 21].

In this study, the Evans index in Turkmen subjects was significantly higher than native Fars subjects.

The *B*/*L* ratio in native Fars subjects was nonsignificantly higher than Turkmen subjects, but the *B*/*H* ratio in Turkmen subjects was nonsignificantly higher than native Fars subjects which means that these parameters were higher than Takeda et al. (2003) in Japanese subjects.

In this study, the Evans index in Turkmen subjects was significantly higher than native Fars subjects. In our results regarding the ethnic groups Evans index in the box were more than Japanese [21].

This study showed evidence for ethnical dimorphism in length of CC, the width of the body, and width of splenium. Our results confirm previous studies which reported racial differences regarding CC parameters [32, 33].

According to Karakaş et al. findings, the size of the widths of Genoa, body, splenium, and height of the corpus callosum were determined to be 13.28 ± 2.10, 7.64 ± 1.07, 12.52 ± 1.35, and 25.47 ± 2.20 mm in females, respectively, whereas, the same measurements were 13.23 ± 2.41, 6.89 ± 2.12, 11.90 ± 1.94, and 25.03 ± 3.38 mm in males, respectively. Due to these findings, Evans ratios were 0.25 ± 1.90 and 0.25 ± 1.14 in females and males, respectively [34].

In the Prendergast et al. study, male subjects were significantly [*F*(1,303) = 6.37, *P* < 0.012] older, on average, than female subjects. There was no handedness significance difference between male subjects [35].

According to Bruner et al. (2012) findings, the differences in measurement and shape of CC between men and women were related to the brain size [36].

Luders et al. (2014) showed the correlation between callosal thickness and brain size in men and women [37].

In contrast, Ardekani et al. (2013) found that the whole CC area was significantly larger in females than males in a linear model, even when matching the male and female participants was done by total brain size [38].

Our previous studies on brain size, head and face size using cephalometry indicated ethnical variation between Turkmen and native Fars people [25–27].

Indeed, according to our previous study, using MRI method in the north of Iran, the size of CC in males was higher than that in females but this difference was not significant, although there was a positive significant correlation between brain longitudinal diameter and length of CC [14].

## 5. Conclusion

This study showed that the corpus callosum parameters vary in different ethnical groups in Gorgan, north of Iran.

## Figures and Tables

**Table 1:** Dimensions of corpus callosum in Iranian male population (Turkmen and native Fars) in north of Iran.

	Mean	95% CI
Age		
Fars (*N* = 40)	36.4	33.4–39.5
Turkmen (*N* = 36)	37.3	33.6–40.96
Width of rostrum		
Fars	11.08	10.58–11.58
Turkmen	11.55	11.04–12.06
Width of splenium		
Fars	11.06^*∗*^	10.70–11.43
Turkmen	11.77^*∗*^	11.25–12.30
Width of body		
Fars	6.38	6.08–6.68
Turkmen	6.87	6.54–7.21
Anterior to posterior length		
Fars	70.21^*∗∗*^	68.85–71.58
Turkmen	74.05^*∗∗*^	72.43–75.68
Height		
Fars	25	24.28–25.74
Turkmen	25.75	24.79–26.71
*B*/*L*		
Fars	0.109	0.072–0.146
Turkmen	0.091	0.084–0.098
*B*/*H*		
Fars	0.256	0.244–0.268
Turkmen	0.27	0.252–0.287
Evans index		
Fars	0.3^*∗∗∗*^	0.295–0.306
Turkmen	0.314^*∗∗∗*^	0.308–0.320

^*∗*^ *P* = 0.033 (difference between Fars and Turkmen males); ^*∗∗*^*P* < 0.0001 (difference between Fars and Turkmen males); ^*∗∗∗*^*P* = 0.001 (difference between Fars and Turkmen males).
